# Simultaneous Determination of Selected Steroids with Neuroactive Effects in Human Serum by Ultrahigh-Performance Liquid Chromatography–Tandem Mass Spectrometry

**DOI:** 10.1021/acschemneuro.3c00824

**Published:** 2024-04-24

**Authors:** Michal Kaleta, Jana Oklestkova, Kateřina Klíčová, Miroslav Kvasnica, Dorota Koníčková, Kateřina Menšíková, Miroslav Strnad, Ondřej Novák

**Affiliations:** † Laboratory of Growth Regulators, Faculty of Science, Palacký University & Institute of Experimental Botany of the Czech Academy of Sciences, Šlechtitelů 27, Olomouc 783 71, Czech Republic; ‡ Department of Neurology, Faculty of Medicine and Dentistry, Palacký University, Olomouc 779 00, Czech Republic; § Department of Neurology, University Hospital Olomouc, Olomouc 779 00, Czech Republic

**Keywords:** neuroactive steroids, neurosteroids, ultrahigh-performance liquid chromatography−tandem mass spectrometry, serum

## Abstract

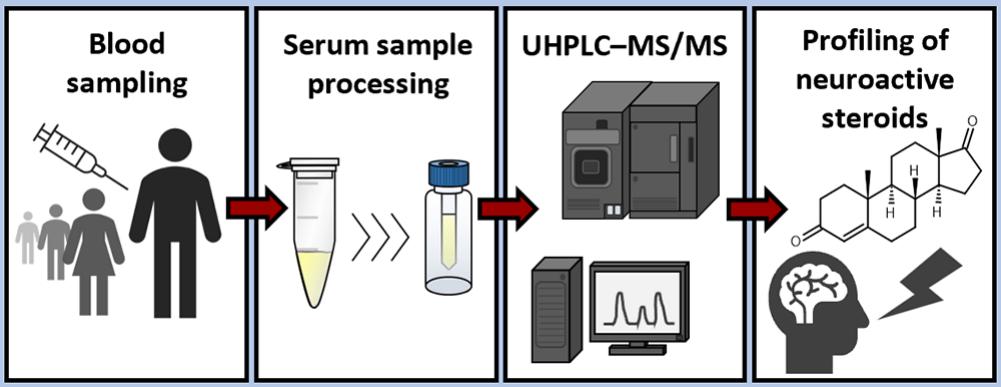

Neuroactive steroids are a group of steroid molecules that are involved in the regulation of functions of the nervous system. The nervous system is not only the site of their action, but their biosynthesis can also occur there. Neuroactive steroid levels depend not only on the physiological state of an individual (person’s sex, age, diurnal variation, etc.), but they are also affected by various pathological processes in the nervous system (some neurological and psychiatric diseases or injuries), and new knowledge can be gained by monitoring these processes. The aim of our research was to develop and validate a comprehensive method for the simultaneous determination of selected steroids with neuroactive effects in human serum. The developed method enables high throughput and a sensitive quantitative analysis of nine neuroactive steroid substances (pregnenolone, progesterone, 5α-dihydroprogesterone, allopregnanolone, testosterone, 5α-dihydrotestosterone, androstenedione, dehydroepiandrosterone, and epiandrosterone) in 150 μL of human serum by ultrahigh-performance liquid chromatography with tandem mass spectrometry. The correlation coefficients above 0.999 indicated that the developed analytical procedure was linear in the range of 0.90 nmol/L to 28.46 μmol/L in human serum. The accuracy and precision of the method for all analytes ranged from 83 to 118% and from 0.9 to 14.1%, respectively. This described method could contribute to a deeper understanding of the pathophysiology of various diseases. Similarly, it can also be helpful in the search for new biomarkers and diagnostic options or therapeutic approaches.

## 1 Introduction

The human nervous system is the source and target tissues for the action of many neuroactive substances. This group undoubtedly includes compounds derived from cholesterol–steroid hormones. All steroids, whether of natural or synthetic origin, that modulate the development and activity of the nervous system and thus the whole organism are referred to as neuroactive steroids (NASs).^1^ They have been observed to be involved in the regulation of neurogenesis, neuritogenesis, synaptogenesis, neuronal survival, myelin formation, synaptic plasticity, and many other processes. Based on these mechanisms, they are involved, for example, in the regulation of mood or behavior. In addition, some of them have a neuroprotective activity that may be sexually dimorphic.^2^ Furthermore, they are involved in learning processes, general activity, memory, and excitatory or inhibitory effects of various neurotransmitter systems.^3^ This large group of steroid substances includes hormonal steroids produced by “typical” steroidogenic peripheral tissues (mainly gonads and adrenal glands) and a specified subgroup of steroids biosynthesized by neurons and glial cells in the central and peripheral nervous system (so-called neurosteroids).^1,2^ The NAS group also includes synthetic steroid substances capable of regulating neural activity. The central and peripheral nervous systems have molecular mechanisms involved in the biosynthesis and metabolism of some NASs. Interestingly, NASs act not only via “classical” intracellular steroid receptors that modulate gene transcription (relatively slow genomic effects) but also through ion channels and membrane receptors (rapid nongenomic effects) such as γ-aminobutyric acid receptors.^3,4^ The nongenomic mechanism of action is typical of NASs and leads to the modulation of neuronal excitability (e.g., γ-aminobutyric acid and *N*-methyl-d-aspartate receptors).^3^ Interactions of NASs with voltage-gated calcium channels, serotonin receptors, voltage-dependent anion channels, microtubule-associated protein 2, etc. are also described. In addition, receptors mediating genomic effects have been directly identified in specific regions of the mammalian brain, including receptors for progesterone, androgens, estrogens, and corticosteroids.^5−8^

In addition to brain imaging methods, various biological markers, especially those of a protein origin (β-amyloid protein, α-synuclein, etc.), are used to predict, prognose, diagnose, or track the progression of neurodegenerative diseases.^9^ Currently, attention is also focused on the possibility of using low-molecular-weight substances.^10,11^ The levels of NASs can also be changed by various pathological events and processes in the nervous system.^1^ For instance, alterations in the levels of some NASs have been observed in psychiatric and neurological diseases such as multiple sclerosis (serum, plasma, and the cerebrospinal fluid),^12,13^ Parkinson’s disease (plasma and the cerebrospinal fluid),^14^ Alzheimer’s disease or non-Alzheimer’s dementia (blood, plasma, brain tissues, and the cerebrospinal fluid),^15,16^ Huntington’s disease (plasma),^17^ and in some injuries such as traumatic brain injury (brain tissues and plasma).^18,19^ In addition to health status, steroidome depends also physiologically on a person’s sex, age, time of sampling (e.g., diurnal variation and menstruation cycle), medication (e.g., oral contraceptive pills in females), dietary patterns, and other factors.^20−24^

The study of steroid substances was initially limited to various arduous colorimetry-based methods or bioassays.^25−27^ The introduction of immunoassays, specifically the radioimmunoassay (RIA), and subsequent modifications of this method marked a major revolution in endocrinology and the analysis of not only steroid hormones.^28,29^ Several platforms based on the RIA and the enzyme immunoassay (especially the enzyme-linked immunosorbent assay, known as ELISA) are available for the determination of steroid analytes.^30−33^ However, immunoassays can have a number of limitations, such as the lack of specificity and associated cross-reactivity with structurally related compounds, limited dynamic range, interference with the matrix, or the ability to analyze only one analyte on a single platform.^34^ In addition to the fact that immunoassays are often unreliable in some cases, for some specialized steroids, they are not even available, so another possible alternative is mass spectrometry (MS). Continuous advances have enabled a wider spread of MS in the bioanalysis of steroid hormones. In recent years, it has become the method of first choice for the determination of steroids.^29^ The use of MS allows highly selective, sensitive, accurate, and precise determination of a large number of steroid analytes in a single analytical run.^35^ For more than half a century, gas chromatography combined with mass spectrometry (GC–MS) has been used for the analysis of steroid substances.^36^ GC-based techniques using single MS or tandem mass spectrometry (MS/MS) are available.^12,37−40^ Approaches to the steroid analysis have been enriched in recent years in particular by the use of liquid chromatography (LC).^13,22,34,41−57^ The LC–MS/MS techniques offer several advantages over GC–MS, such as the high-throughput analysis (more suitable for a large set of samples), less time-consuming and generally easier sample preparation (usually, no derivatization is required), or the ability to quantify intact steroid conjugates (sulfates or glucuronides).^58^ These properties make LC–MS/MS more suitable for routine use in clinical laboratories. Reversed-phase chromatographic separation is widely applied in the analysis of steroid substances. These are stationary phases based on hydrocarbon chains of various lengths (mainly C18),^22,41−57^ phenyl-hexyl^44^ or, for example, pentafluorophenyl.^34^ Detection is most commonly provided by triple-quadrupole MS,^22,34,41,43,45−50,52−56^ but various hybrid approaches are also available (e.g., linear ion trap with an Orbitrap or triple-quadrupole with a linear ion trap).^42,44,51,57^ NASs are studied by LC–MS/MS in various types of mammalian biological matrices such as plasma or serum,^13,22,34,41,43,47−49^ the cerebrospinal fluid,^13,31,43^ and brain tissues.^47,50,51^ However, others can also be used: urine,^52,53^ saliva,^42,54,55^ hair,^46,56^ or nails.^57^ LC–MS/MS-based methods have been successfully used to determine selected representatives of androgens, estrogens, progestins, and corticosteroids.^59^

Even when the best possible end point analytical approach is available, sample processing is still a critical step in the analysis. Sample preparation for the LC–MS/MS analysis of low-abundance endogenous compounds in complex biological matrices usually involves protein precipitation (e.g., with acetonitrile and methanol/water containing zinc sulfate),^34,41,44,54^ solid-phase extraction (SPE; online or offline),^13,22,42,44−46,48,51−54,56,57^ or liquid–liquid extraction (LLE)^34,43,46,47,49,53,55,57^ in various combinations and modifications. In addition, various derivatization procedures can be used to improve detection capabilities (e.g., with dansyl chloride, hydroxylamine hydrochloride, or picolinic acid).^34,43,49−51,56^ Enzymatic hydrolysis can also be part of the sample preparation for urinary steroid analysis.^53^ However, it is important to note that the design of the purification process depends on the specific analytical requirements (e.g., the type and quantity of the sample and the purpose of the analysis).

**Figure 1:**
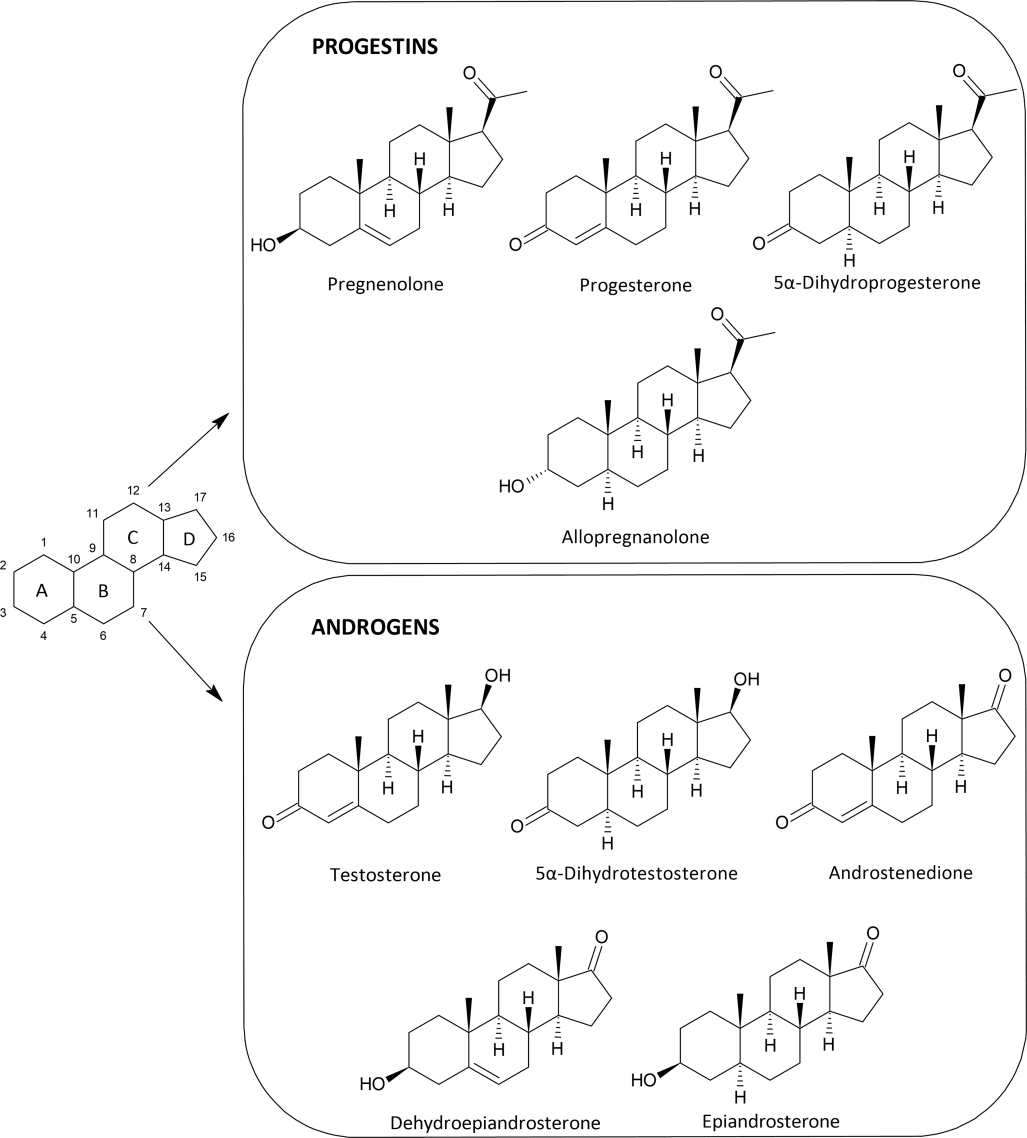
Structures of the analyzed steroid hormones.

The aim of this work was to develop and validate a comprehensive isotope dilution method for a simultaneous analysis of selected NASs, such as the progestins pregnenolone (PREG), progesterone (PROG), 5α-dihydroprogesterone (DHP), and allopregnanolone (ALLO) and the androgens dehydroepiandrosterone (DHEA), androstenedione (ANDRO), testosterone (T), 5α-dihydrotestosterone (DHT), and epiandrosterone (EPIA) in human blood serum. The chemical structures of the individual analytes and their physicochemical properties are shown in Figure 1 and Table S1, respectively. These analytes are very important representatives of female and male steroid hormones and have been also studied in other NAS-focused research.^12−14,31,47^ The developed method combines a very simple and rapid extraction process with sensitive detection and quantification by ultrahigh-performance liquid chromatography with electrospray tandem mass spectrometry (UHPLC–ESI–MS/MS). To the best of our knowledge, no rapid and high-throughput comprehensive method is currently available for the simultaneously targeted profiling of a selected set of steroids with neuroactive effects in human serum. Due to numerous pieces of evidence of alterations in neuroactive steroid levels in several pathologies of the nervous system (see above), the use of this method offers considerable potential. In addition, the discovery of reliable serum biomarkers is still a major challenge.^60^ Importantly, the application of the developed method and its ability to profile nine NASs in a single analysis from relatively easy-to-obtain blood serum samples in micromolar quantities increase the efficiency of new biomarker discovery. In general, the use of robust analytical approaches has the potential to detect metabolic changes and patterns specific to certain pathologies. The acquired knowledge may be potentially helpful, for example, in the differential diagnosis of specific pathologies. Moreover, the profiling of NASs may also guide the dosing and monitoring of the efficacy of treatments that modulate NAS levels. The potentially therapeutically beneficial effects of the analytes investigated are summarized in Table 1.

**Table 1:** Selected Potential Therapeutic Targets

**therapeutic target**	**function**	**references**
pregnenolone	precursor to other steroids; effects on anxiety, cognition, memory, etc.	(61)
	neuroprotective activity	(62)
progesterone	neuroprotective effect	(63,64)
	antitumor activity	(65)
5α-dihydroprogesterone	neuroprotective effect	(64)
allopregnanolone	neuroprotective effect	(63)
	anticonvulsant properties	(66)
	antidepressant-like effect	(67)
	analgesic effects	(68)
dehydroepiandrosterone	modulation of endothelial function, reduction of inflammation, neuroprotective activity, improved cognitive function, memory enhancement, etc.	(69)
androstenedione	neurosteroid, antiseizure capacity, modulation of the glutamate receptor and voltage-gated Ca^2+^ channels	(70)
testosterone	anabolic properties and antiseizure capacity	(70)
5α-dihydrotestosterone	antineuroinflammatory and neuroprotective effects	(71)
epiandrosterone	antiviral activity	(72)
	anabolic properties; a precursor of 7β-hydroxy-epiandrosterone that exhibits neuroprotective, anti-inflammatory, and antiestrogenic effects	(73)

## 2 Results and Discussion

### 2.1 Chromatographic Separation and Mass Spectrometric Detection

**Figure 2:**
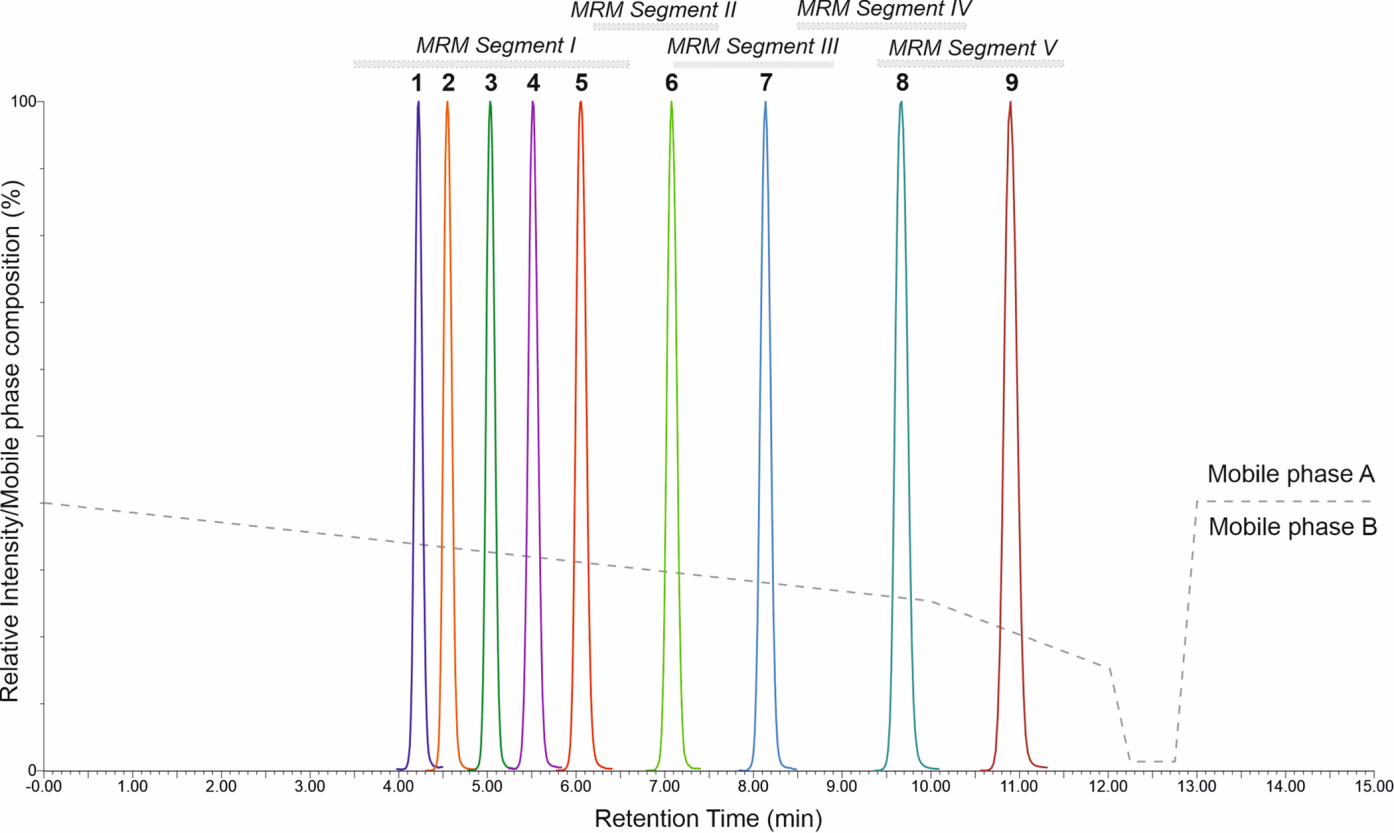
Reversed-phase chromatographic separation of nine steroid standards dissolved in 100% methanol (900 nmol/L): dehydroepiandrosterone (1), testosterone (2), epiandrosterone (3), 5α-dihydrotestosterone (4), androstenedione (5), pregnenolone (6), allopregnanolone (7), progesterone (8), and 5α-dihydroprogesterone (9). The chromatographic run is divided into five multiple-reaction monitoring (MRM) scan segments.

A high percentage of published methods rely on reversed-phase chromatographic columns,^22,42,44−46,48,49,55,56,74−76^ and therefore, this strategy was chosen in this case. As is well-known, retention during reversed-phase separation is usually based mainly on hydrophobic and van der Waals interactions.^77^ Therefore, to improve chromatographic selectivity, we employed a biphenyl LC column, known for its suitability in the dosage of steroids.^78^ The retention mechanisms primarily rely on hydrophobic and π–π interactions in the presence of methanol (MeOH). Nine target steroids were successfully separated at the baseline using a Kinetex biphenyl column (100 × 2.1 mm, 1.7 μm, 100 Å; Phenomenex, USA) equipped with an ACQUITY column in-line filter kit (Waters) (Figure 2). Retention times (RTs) of the analytes studied ranged from 4.16 (DHEA) to 10.83 min (DHP). A high degree of RT stability was observed for all compounds during the UHPLC–MS/MS analysis. The maximum standard deviation (SD) of RTs between injections (*n* = 60) was 0.03 min (Table 2). The coefficient of variation (CV) values ranged from 0.29 to 0.57% for all substances tested.

**Table 2:** Summary of Multiple-Reaction Monitoring Transitions, Optimized Instrument Settings, and Retention Times (min; Means ± SD; *n* = 60) for Individual Analytes and Internal Standards^a^

**analyte/internal standard**	**RT ± SD** (min)	**precursor ion**	**molecular weight** (g/mol)	**quantif. MRM transition**	**confirm. MRM transition**	**cone voltage** (V)	**collision energy** (eV)	**dwell time** (ms)
MRM scan segment I(3.5–6.7 min)								
DHEA	4.16 ± 0.02	[M – H_2_O + H]^+^	288	271 > 253	271 > 213	20/27	13/16	0.050
*d*_6_-DHEA	4.10 ± 0.02	[M – H_2_O + H]^+^	294	277 > 259		29	13	0.050
T	4.49 ± 0.02	[M + H]^+^	288	289 > 97	289 > 109	25/22	19/22	0.050
*d*_3_-T	4.45 ± 0.03	[M + H]^+^	291	292 > 97		25	19	0.050
EPIA	4.97 ± 0.02	[M – H_2_O + H]^+^	290	273 > 255	273 > 161	30/30	11/18	0.050
DHT	5.45 ± 0.03	[M + H]^+^	290	291 > 255	291 > 159	30/30	13/21	0.050
ANDRO	5.98 ± 0.03	[M + H]^+^	286	288 > 97	288 > 109	20/20	18/21	0.050
MRM scan segment II(6.2–7.6 min)								
PREG	7.00 ± 0.03	[M – H_2_O + H]^+^	316	317 > 159	317 > 81	25/25	22/25	0.095
*d*_4_-PREG	6.91 ± 0.03	[M – H_2_O + H]^+^	320	321 > 159		25	22	0.095
MRM scan segment III(7.1–8.9 min)								
ALLO	8.06 ± 0.03	[M – H_2_O + H]^+^	318	301 > 81	301 > 95	45/45	30/25	0.225
*d*_4_-ALLO	7.97 ± 0.03	[M – H_2_O + H]^+^	322	305 > 81		45	30	0.225
MRM scan segment IV(8.5–10.4 min)								
PROG	9.58 ± 0.03	[M + H]^+^	314	315 > 97	315 > 109	23/25	19/22	0.250
*d*_9_-PROG	9.40 ± 0.03	[M + H]^+^	323	324 > 100		19	21	0.250
MRM scan segment V(9.9–11.5 min)								
DHP	10.83 ± 0.03	[M + H]^+^	316	317 > 85	317 > 281	32/30	13/13	0.240

a ALLO: allopregnanolone, ANDRO: androstenedione, *d*_3_-T: testosterone-*d*_3_, *d*_4_-ALLO: allopregnanolone-*d*_4_, *d*_4_-PREG: pregnenolone-*d*_4_, *d*_6_-DHEA: dehydroepiandrosterone-*d*_6_, *d*_9_-PROG: progesterone-*d*_9_, DHEA: dehydroepiandrosterone, DHP: 5α-dihydroprogesterone, DHT: 5α-dihydrotestosterone, EPIA: epiandrosterone, MRM: multiple-reaction monitoring, PREG: pregnenolone, PROG: progesterone, RT: retention time, SD: standard deviation, and T: testosterone.

Analyte profiling was performed using a triple-quadrupole with positive electrospray ionization (ESI^+^) in multiple-reaction monitoring (MRM) acquisition mode. The MRM mode is characterized by exceptional selectivity and sensitivity in ion recording methods and is therefore widely used for the quantification of low-abundance target analytes.^79^ Increased reliability in the quantification of analytes was achieved by combining the UHPLC–MS/MS approach with the stable isotope dilution method.^80^ Two MRM transitions were monitored for each analyte. The more intense mass transition was used as a quantification transition, and the other was used as a confirmation transition (Table 2). Importantly, the selection of MRM transitions of individual analytes and labeled standards was verified on the basis of existing publications using the LC–MS/MS system for the analysis of steroid compounds.^74−76,81−85^ Precursor ions were either protonated [M + H]^+^ molecules or [M – H_2_O + H]^+^ molecules formed by the loss of water molecules due to instability in the ESI source (see Table 2). In addition, the cone voltage and especially the collision energy for each MRM transition have been also optimized to achieve the highest possible sensitivity. The values that showed the largest peak area in the UHPLC–MS/MS measurements were selected and are reported in Table 2. The optimized cone voltage and collision energy values ranged from 20 to 45 V and 11 to 30 eV, respectively. To achieve optimal sensitivity, the chromatographic window was divided into five MRM scan segments based on the expected RTs of the analytes. Dwell time values were set between 0.050 and 0.250 ms to achieve at least 15 scan points per chromatographic peak.

### 2.2 Method Calibration

Quantification of the analytes was performed using matrix surrogate calibration curves (4% bovine serum albumin in 10 mmol/L phosphate-buffered saline (PBS) buffer). An important step in their construction was the determination of the parameters that characterize them, namely, the linear range of the calibration curve, the limit of detection (LOD), the lower limit of quantification (LLOQ), and the upper limit of quantification (ULOQ). The LLOQ is defined as a signal-to-noise ratio (*S/N*) ≥ 5 and quantified with acceptable accuracy and precision.^86^ LLOQ is represented by the lowest concentration point in the calibration range.^86^ The LOD values (*S/N* = 3) were estimated by knowledge of the signal-to-noise ratio of the LLOQ points.

Overall, calibration parameters for all target analytes are listed in Table 3. The LLOQ values of selected steroids in the surrogate matrix ranged from 0.90 to 28.46 nmol/L. The lowest LLOQ values in the blank matrix were obtained for T, ANDRO, and PROG (0.90 nmol/L), while the highest was obtained for DHP (28.46 nmol/L). In addition, some analytes (T, ANDRO, and PROG) can be detected at concentrations lower than 0.36 nmol/L. Such low values allowed for the profiling of analytes at the trace level in serum samples (see below). Based on the available data, the LLOQ values achieved for the vast majority of analytes were at or close to the expected endogenous levels of the target analytes.^87^ It should be noted that lower LLOQ and LOD values can of course be achieved using the calibration curves prepared in a pure solvent. However, it is recommended to use the matrix surrogate calibration curve for the analysis of metabolites in biological samples.^86^ Compared to many available studies, LLOQ values for T,^34,39,47,81,82,85^ DHEA,^35,39,41,45,47,81,82,85^ PROG,^35,39,41,44,47^ PREG,^39,47^ ANDRO,^39,41,81,82,85^ ALLO,^88,89^ and DHT^39^ were better or at least within 1 order of magnitude. Lower quantification limits for some substances in other methods may be due to the use of different ionization techniques.^41^ One strategy that can potentially reduce the matrix effects (MEs) observed in LC–MS involves the careful selection of the ionization technique.^90^ ESI, atmospheric-pressure chemical ionization (APCI), and atmospheric-pressure photoionization (APPI) are frequently employed for steroid analysis (in this order), with the latter two exhibiting notably reduced MEs.^91^ This observation is attributed to their higher selectivity in ionization processes. The ability to ionize all steroids and even estrogens in positive polarity can also be considered an advantage of APPI.^92^ Due to their sensitivity and ability to reduce MEs, APPI and APCI ionization techniques appear to be more suitable for steroid analysis.^93^ However, conclusions regarding whether the APCI or APPI technique is more suitable for steroid analysis are not consistent.^94,95^ However, none of these techniques were available in our laboratory, and therefore, the ESI source was used. Furthermore, the negative effects of the biological matrix (coeluting components) can also be significantly reduced by appropriately selected sample preparation involving various purification procedures (precipitation, LLE, SPE, etc.).^90^ In general, LLE and SPE approaches provide much purer extracts and may lead to less matrix influence compared to simple protein precipitation, which does not allow the removal of salts and phospholipids.^96^ However, there are commercial precipitation platforms that can accomplish this. Moreover, some of the more sophisticated methods inevitably require specialized laboratory equipment and increase the processing time, cost, and complexity of sample preparation. Yuan et al. achieved better LLOQ values for many analytes (PREG, DHEA, ANDRO, and PROG), but their sample preparation involves derivatization steps, specifically acylation with isonicotinoyl chloride.^34^

**Table 3:** Summary of Linear Ranges, Detection and Quantification Limits, and Regression Parameters of Serum Matrix Surrogate Calibration Curves^a^

				**regression parameters**		
**analyte**	**linear range**^b^	**LOD**^b^	**LLOQ**^b^	**slope**	**intercept**	***r***^**2**^
DHEA	9.00–28,460.50	4.12	9.00	0.9991	0.3067	0.9996
T	0.90–28,460.50	0.14	0.90	0.9836	0.1294	0.9996
EPIA	9.00–28,460.50	2.19	9.00	1.0355	–0.7937	0.9998
DHT	2.85–28,460.50	1.04	2.85	1.0247	–0.6112	0.9996
ANDRO	0.90–28,460.50	0.29	0.90	0.9979	–0.1018	0.9996
PREG	2.85–28,460.50	1.27	2.85	1.0072	–0.1642	0.9997
ALLO	9.00–28,460.50	3.15	9.00	0.9993	0.2419	0.9995
PROG	0.90–9000.00	0.36	0.90	0.9973	0.3037	0.9996
DHP	28.46–28,460.50	11.91	28.46	0.9639	–1.2306	0.9989

a ALLO: allopregnanolone, ANDRO: androstenedione, DHEA: dehydroepiandrosterone, DHP: 5α-dihydroprogesterone, DHT: 5α-dihydrotestosterone, EPIA: epiandrosterone, LLOQ: lower limit of quantification (S/*N* ≥ 5), LOD: limit of detection (S/*N* = 3), PREG: pregnenolone, PROG: progesterone, and T: testosterone.

b nmol/L for all analytes.

A minimum seven-point matrix surrogate calibration curve was obtained for all analytes tested. To assess linearity, the analyte concentration was back-calculated for each point on the calibration curve and related to the nominal concentration of that point. The difference between the calculated value and the nominal value did not exceed ±15%, in the case of LLOQs ±20%.^86^ In constructing the calibration curves, each calibration point was interleaved with a linear regression line. Individual calibration curves are defined by the line equation (slope and intercept) and by the coefficient of determination (*r*^2^). Linearity was excellent, with *r*^2^ varying between 0.9989 and 0.9998 for all analytes tested (Table 3).

The analysis of a solvent sample (100% MeOH) beyond the most concentrated point (ULOQ) of the calibration curve confirmed no significant carryover between samples. This confirms that there is no interference between samples (even between the samples with high concentrations of analytes) that would interfere with the analysis.

### 2.3 Method Precision and Accuracy

The within-run and between-run precision and accuracy of the analytical method were determined using four sets of neat solution samples, each set spiked to one quality control (QC) concentration level (low QC, LQ; medium QC, MQ; high QC, HQ; ultrahigh QC, UHQ). Each QC level was represented by five samples. The accuracy is expressed as a percentage and represents the closeness of the measured concentration to the reference value.^86^ In contrast, the precision of the method is expressed as a CV. To determine the between-run parameters, the same set of samples was analyzed in three different analytical runs on two different days. The precision and accuracy values for selected steroid analytes in a solvent are shown in Table 4.

**Table 4:** Within-Run and Between-Run Precision and Accuracy at Low (LQ), Medium (MQ), High (HQ), and Ultrahigh (UHQ) Levels of Steroid Analytes in Neat Solutions^a^

	**accuracy** mean (%) (SD)								**precision** CV (%)							
	within-run				between-run				within-run				between-run			
QC levels^b^	LQ	MQ	HQ	UHQ	LQ	MQ	HQ	UHQ	LQ	MQ	HQ	UHQ	LQ	MQ	HQ	UHQ
DHEA	100 (4.3)	102 (4.3)	114 (2.1)	102 (1.8)	100 (3.7)	101 (3.2)	114 (1.5)	102 (1.7)	4.4	4.2	1.8	1.8	3.7	3.2	1.3	1.7
T	97 (1.1)	97 (1.1)	110 (1.1)	96 (0.9)	97 (1.3)	98 (1.1)	110 (0.9)	95 (1.1)	1.1	1.1	1.0	0.9	1.3	1.2	0.8	1.1
EPIA	94 (4.7)	91 (4.9)	103 (5.8)	94 (5.7)	95 (3.3)	93 (4.5)	105 (3.8)	95 (6.4)	5.0	5.4	5.6	6.0	3.5	4.8	3.6	6.8
DHT	98 (4.4)	95 (2.9)	105 (3.3)	96 (4.0)	97 (3.2)	95 (2.8)	106 (2.1)	95 (4.6)	4.5	3.1	3.1	4.2	3.3	2.9	2.0	4.8
ANDRO	101 (1.0)	98 (1.4)	110 (0.7)	99 (1.2)	100 (1.2)	97 (1.4)	110 (0.8)	98 (1.5)	0.9	1.4	0.6	1.2	1.2	1.4	0.8	1.5
PREG	97 (5.7)	97 (1.6)	111 (0.6)	98 (0.4)	97 (3.7)	98 (1.7)	112 (1.0)	98 (0.5)	5.9	1.7	0.6	0.4	3.8	1.7	0.9	0.5
ALLO	93 (1.7)	93 (0.9)	112 (0.7)	100 (0.6)	94 (2.4)	93 (0.9)	112 (0.8)	100 (0.6)	1.8	0.9	0.6	0.6	2.5	0.9	0.7	0.6
PROG	95 (0.4)	98 (1.0)	112 (1.1)	95 (0.2)	95 (0.8)	98 (0.9)	112 (0.7)	94 (0.5)	0.4	1.0	1.0	0.2	0.8	1.0	0.7	0.6
DHP	96 (2.7)	98 (1.5)	113 (1.2)	102 (0.8)	95 (2.9)	98 (1.9)	114 (2.2)	102 (2.1)	2.8	1.5	1.1	0.8	3.0	2.0	2.0	2.1

a ALLO: allopregnanolone, ANDRO: androstenedione, DHEA: dehydroepiandrosterone, DHP: 5α-dihydroprogesterone, DHT: 5α-dihydrotestosterone, EPIA: epiandrosterone, PREG: pregnenolone, PROG: progesterone, and T: testosterone. Accuracy was expressed as a percentage of the nominal concentration; *n* = 5. Precision was expressed as a coefficient of variation (CV); *n* = 5.

b The low (LQ), medium (MQ), high (HQ), and ultrahigh (UHQ) levels correspond to 28.46, 90, 900, and 2846.05 nmol/L, respectively.

Both the within-run and between-run accuracy fell within ±15% for all of the steroids. This is consistent with the European Medicine Agency (EMA) requirements.^86^ The lowest within-run and between-run accuracy was determined for EPIA (91%) and the highest for DHEA and DHP (114%). The SD values were below 10% for all steroid analytes. The requirements were also met in the case of method precision. Specifically, CVs ranged between 0.2% for PROG and 6.8% for EPIA (both at the UHQ level).

Furthermore, the accuracy and precision of the analytical method were evaluated using the pooled spiked serum (see Table 5). Each QC level (LQ, MQ, HQ, and UHQ) was represented by five samples. To determine the accuracy of the method, mean analyte concentrations were compared to nominal values. For all analytes, 83 to 118% was achieved, indicating the reliability and accuracy of the method. The measured mean concentrations did not deviate from the reference values by more than ±15% (20%). The accuracy of the developed method is therefore in accordance with the requirements set by the EMA.^86^ The CV values also reach the required values (from 0.9 to 14.1%). Based on these results, it can be concluded that the laboratory and systematic errors of the method are not significant.

**Table 5:** Within-Run Precision and Accuracy at Low (LQ), Medium (MQ), High (HQ), and Ultrahigh (UHQ) Levels of Steroid Analytes in Human Serum^a^

	**accuracy** mean (%) (SD)				**precision** CV (%)			
**QC levels**^b^	LQ	MQ	HQ	UHQ	LQ	MQ	HQ	UHQ
DHEA	87 (4.7)	94 (1.2)	105 (1.4)	106 (4.0)	5.4	1.2	1.3	3.8
T	87 (2.1)	90 (2.3)	100 (0.9)	100 (3.3)	2.4	2.6	0.9	3.2
EPIA	117 (3.0)	106 (12.1)	115 (1.1)	102 (14.4)	2.6	11.3	1.0	14.1
DHT	86 (2.6)	85 (1.5)	92 (0.9)	86 (9.2)	3.0	1.8	0.9	10.7
ANDRO	83 (1.7)	84 (1.5)	93 (0.9)	93 (5.9)	2.1	1.8	1.0	6.3
PREG	96 (2.3)	99 (3.3)	108 (1.8)	108 (4.3)	2.4	3.3	1.7	4.0
ALLO	110 (12.0)	109 (4.9)	115 (2.6)	115 (5.7)	10.9	4.5	2.3	4.9
PROG	100 (3.7)	105 (3.0)	114 (2.3)	114 (2.8)	3.7	2.9	2.0	2.4
DHP	110 (7.3)	102 (2.7)	115 (1.0)	118 (4.2)	6.6	2.7	0.9	3.6

a ALLO: allopregnanolone, ANDRO: androstenedione, DHEA: dehydroepiandrosterone, DHP: 5α-dihydroprogesterone, DHT: 5α-dihydrotestosterone, EPIA: epiandrosterone, PREG: pregnenolone, PROG: progesterone, and T: testosterone. Accuracy was expressed as a percentage of the nominal concentration; *n* = 5. Precision was expressed as a coefficient of variation (CV); *n* = 5.

b The low (LQ), medium (MQ), high (HQ), and ultrahigh (UHQ) levels correspond to 28.46, 90, 900, and 2846.05 nmol/L, respectively.

### 2.4 Method Recovery and Matrix Effects

**Figure 3:**
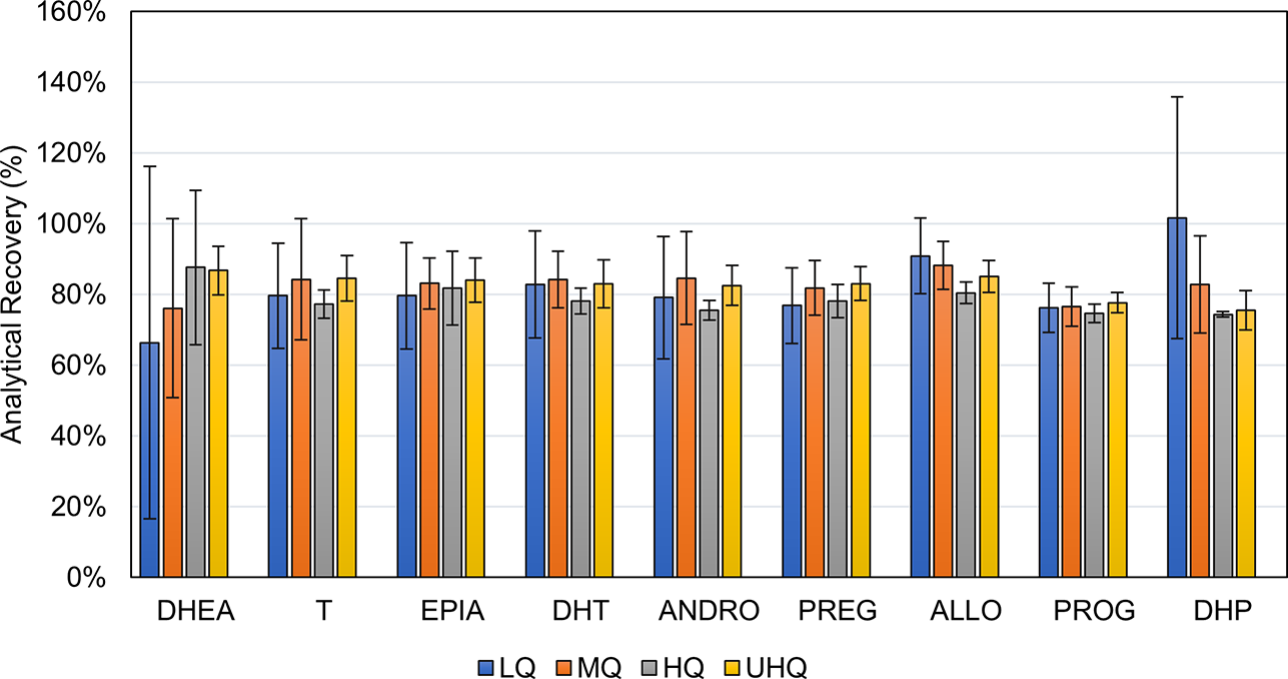
Analytical recovery of selected steroid analytes in four lots of serum at low (LQ)-, medium (MQ)-, high (HQ)-, and ultrahigh (UHQ)-quality control levels (*n* = 12). The abbreviations of the analytes are given in the list of abbreviations.

The recovery (RE) of the method was tested at four concentration levels (corresponding to LQ, MQ, HQ, and UHQ) by using blood serum from several donors. For the RE determination, the spiked serum samples before and after extraction were compared; the calculation was based on Matuszewski et al.^97^ The analytical method REs ranged from 66 to 102% (Figure 3). The greatest losses during the purification and extraction process occur with DHEA at the LQ level. However, these results generally indicate efficient extraction of target analytes from serum samples. The higher SDs (from 1 to 50%) can be explained by the use of four lots of blood serum from different donors. Thus, it can be concluded that the RE in this case is highly dependent on the individual characteristics of the samples. Interestingly, hemolysis, icterus, paraproteinemia, and lipemia, for example, can interfere with biochemical tests.^98^ We hypothesize that a similar effect, i.e., different extraction efficiency of analytes due to differences in the matrix (e.g., increased hemoglobin, lipid content, etc.), is also possible in this case. The sample preparation of the developed method is relatively simple and rapid; practically, it only involves precipitation of serum proteins, filtration, and concentration. Despite this simplicity, none of the analytes showed a decrease in method RE below 66%. Compensation for these sample processing losses is provided by the use of a defined addition of stable isotopically labeled ISs.^99^ In fact, sample processing for many steroid analysis methods usually includes additional steps using, for example, solid-phase extraction^13,22,42,45,46,48,51−54,56^ or even derivatization.^34,43,49−51,56^ The elimination of other usually time-consuming steps makes this method high-throughput and relatively cost-effective.

Another important validation parameter is ME, which can negatively affect the accuracy, precision, or sensitivity of the analytical method.^90,97^ A set of blood serum samples spiked after extraction to four QC levels were used for its determination. In this study, the values of absolute ME of the analyzed steroids at all QC levels ranged between 19 (for DHP) and 117% (for DHEA) (Table 6). In addition, the IS-normalized ME was also determined,^100^ with a maximum CV of 14.4%, which is in accordance with EMA guidelines.^86^ For most steroid analytes, the ME is effectively compensated for by the ISs used. These results confirm that in addition to matrix-matched (alternatively surrogate) calibration curves and optimization of sample preparation, chromatography, and mass spectrometry, ISs (structural analogues or stable isotope-labeled compounds) can be used to remove or at least reduce ME.^90^ The use of ISs increases the robustness of the developed method. The strongest absolute and IS-normalized ME in terms of ion suppression was observed for DHP, specifically from 19 to 24% and from 27 to 33%, respectively. DHP has the highest log *P* value of all the analytes tested, i.e., it is the least polar analyte and has the highest retention on the biphenyl stationary phase of the LC column (Table S1 and Figure 2). Its elution is due to the increasing concentration of an organic solvent in the mobile phase (i.e., decreasing polarity). We assume that such a strong ME is due to the elution of DHP at the end of the gradient, together with a high proportion of contaminants. Matrix components such as peptides, lipids, salts, or urea that elute together with the analyte can interfere with the efficiency of its ionization, either by ion enhancement or, in this case, ion suppression.^90,97^ Additionally, phospholipids often cause ME in the analysis of plasma or serum using LC–MS/MS methods.^101^ The reason for the lack of compensation of the ME by the IS may be due to the fact that progesterone-*d*_9_ (*d*_9_-PROG) was used, which elutes at a different RT than that of DHP and therefore in an environment containing different interfering substances. Unfortunately, a stable isotopically labeled analogue of DHP was not available in our laboratory. Nevertheless, the developed method allows reliable quantification of DHP, which was confirmed in accuracy and precision testing on blood serum samples. This correction is provided by a matrix surrogate calibration curve that is subjected to the same purification and extraction protocol as real samples.

**Table 6:** Evaluation of the Matrix Effect in Human Serum^a^

	**absolute ME** (SD)^b^				**IS-normalized ME** (CV)^b^			
**analyte**	**LQ**	**MQ**	**HQ**	**UHQ**	**LQ**	**MQ**	**HQ**	**UHQ**
DHEA	117 (13.9)	99 (10.9)	100 (15.8)	103 (7.3)	99 (4.5)	100 (5.0)	91 (4.1)	99 (3.1)
T	93 (9.7)	98 (1.8)	98 (7.0)	97 (2.7)	89 (6.9)	90 (4.4)	81 (4.9)	88 (3.8)
EPIA	109 (3.5)	109 (7.7)	109 (9.0)	111 (4.2)	101 (11.3)	101 (3.6)	91 (14.4)	100 (6.6)
DHT	93 (2.4)	90 (4.2)	91 (5.3)	91 (4.5)	86 (10.5)	83 (1.6)	75 (13.6)	82 (7.4)
ANDRO	93 (4.9)	98 (4.8)	102 (1.0)	101 (5.2)	87 (10.1)	90 (3.4)	85 (9.6)	91 (5.7)
PREG	113 (2.4)	113 (5.9)	115 (3.2)	116 (3.4)	96 (2.3)	99 (3.5)	91 (4.7)	99 (3.8)
ALLO	71 (2.4)	71 (3.9)	70 (0.5)	71 (1.6)	113 (4.7)	115 (3.6)	100 (2.8)	107 (2.3)
PROG	74 (2.8)	78 (3.6)	76 (1.9)	79 (2.8)	101 (3.2)	106 (3.2)	97 (3.8)	109 (3.2)
DHP	19 (2.7)	22 (1.8)	24 (1.3)	24 (1.5)	27 (13.3)	30 (4.9)	30 (7.5)	33 (4.8)

a ALLO: allopregnanolone, ANDRO: androstenedione, DHEA: dehydroepiandrosterone, DHP: 5α-dihydroprogesterone, DHT: 5α-dihydrotestosterone, EPIA: epiandrosterone, PREG: pregnenolone, PROG: progesterone, and T: testosterone.

b The matrix effect (ME), standard deviation (SD), and coefficient of variation (CV) are expressed in percentage. These values were calculated from 4 lots of matrices tested in triplicates (*n* = 12). The low (LQ), medium (MQ), high (HQ), and ultrahigh (UHQ) levels correspond to 28.46, 90, 900, and 2846.05 nmol/L, respectively.

### 2.5 Stability of the Analytes

The stability of the analytes can be affected during any sample handling step (aliquoting, extraction procedures, evaporation, reconstitution, etc.).^102^ The storage temperature, enzyme activity, or, for example, the pH of the samples can play a role. Current literature focusing comprehensively on the stability of some steroid substances is very limited. Serum levels of androgens such as T and DHT still show high reproducibility after 10 years of storage at −80 °C without thawing.^103^ Their concentrations were not significantly altered even after multiple freeze–thaw cycles. The steroid hormones T, PREG, PROG, ANDRO, and DHEA should be stable in blood serum for at least 72 h at 25 °C, 7 days at 4 °C, and 60 days at −80 °C.^34^ The concentrations of these analytes did not differ from the theoretical values by more than 10% even after three cycles of thawing (25 °C) and freezing (−80 °C). The stability of some steroids during storage can be affected by the addition of anticoagulants (e.g., ethylenediaminetetraacetic acid).^102,104^

Our results show that the vast majority of the tested steroids were sufficiently stable (differences of less than 10%) in neat solution, pooled serum, and surrogate matrix when stored in an autosampler at 4 °C for 7 days (Tables S2, S3, and S4). The largest difference was observed for DHP, where the variation detected in pooled serum ranged from 6 to 60%. This may be due to the use of an uncorresponding internal standard (*d*_9_-PROG) and a strong ME (as discussed above). Importantly, for the time required to analyze a fully filled autosampler (i.e., approximately 24 h for 96 samples), the stability of DHP was sufficient.

### 2.6 Profiling of Steroid Analytes in Serum

Finally, the validated UHPLC–ESI–MS/MS method was applied to steroid analysis in a selected group of participants. A total of 16 donors with different types of nervous system pathologies were included in this study. Each sample was represented by a triplicate. The participant group consisted of 8 males aged 41–67 years (median 57.5 years) and 8 females aged 21–51 (median 35.5 years). The median and range of the determined endogenous levels of individual analytes are listed in Table 7.

**Table 7:** Endogenous Levels of Target Steroids in Donor Serum (*n* = 16)^a^

	**median (range)** (nmol/L)			
**analyte**	**female** (*n* = 8)	**male** (*n* = 8)	**observed serum/plasma circulating levels** (nmol/L)	**references**
DHEA	16.16 (6.13–29.23)	7.22 (4.16–18.61)	4.13–65.69 (F-premenopausal)	(105)
			4.86–49.55 (M)	
T	1.50 (0.57–3.59)	8.53 (4.50–30.30)	0.36–1.57 (F-premenopausal)	(105)
			9.78–28.36 (M)	
EPIA	2.00 (2.00–3.92)	2.62 (2.00–6.01)	0.33–0.57 (F-menstrual cycle/pregnancy)	(37)
			0.31 (M)	
DHT	0.63 (0.63–1.45)	1.05 (0.63–3.38)	0.09–0.91 (F-follicular phase)	(106,107)
			0.38–3.27 (M)	
ANDRO	3.76 (2.30–5.28)	2.42 (2.04–4.32)	0.97–5.72 (F-premenopausal)	(105)
			0.92–4.41 (M)	
PREG	3.62 (1.97–6.64)	2.23 (0.63–3.83)	0.54–4.11 (F-premenopausal)	(108)
			0.88–5.21 (M)	
ALLO	2.00 (2.00–2.00)	2.00 (2.00–2.00)	<1–5/<157 (F-menstrual cycle/pregnancy)	(109−111)
			<1 (M)	
PROG	1.44 (0.20–34.82)	0.20 (0.20–0.33)	<56.66 (F-premenopausal)	(105)
			<0.60 (M)	
DHP	6.32 (6.32–6.32)	6.32 (6.32–10.52)	0.19–31.0 (F-menstrual cycle/pregnancy)	(112,113)
			mean 55.04 ± 24.84 (M)	

a ALLO: allopregnanolone, ANDRO: androstenedione, DHEA: dehydroepiandrosterone, DHP: 5α-dihydroprogesterone, DHT: 5α-dihydrotestosterone, EPIA: epiandrosterone, PREG: pregnenolone, PROG: progesterone, T: testosterone, M: male, and F: female.

The measured serum concentrations of the analytes in samples of 8 males and 8 females were in most cases consistent with endogenous levels found in presumably healthy volunteers in other studies (Table 7). Obviously, there are some concentration differences depending on the analytical techniques used, the size and composition of the cohorts tested, the method of sampling, and many other factors. The levels of these analytes naturally vary depending on the sex and age of the individual but also during various physiological changes in the human body (e.g., menstrual cycle and pregnancy). These factors, including any treatment specifically modifying steroid levels, should be strictly considered when designing future studies in steroidomics. Missing analyte levels were replaced by two-thirds of the respective LOQ values^114^ (9 values for EPIA, 7 values for DHT, 2 for PREG, 16 for ALLO, 7 for PROG, and 15 for DHP). It is important to note that the samples were concentrated several times during the purification and extraction process. Nevertheless, the determination of the lowest endogenous levels in human serum was difficult. However, the developed method can be reliably applied to some physiological conditions in which natural levels increase several-fold. For example, the level of ALLO fluctuates in women of reproductive age from less than 1 to 5 nmol/L (depending on the phase of the menstrual cycle); at the end of the third trimester of pregnancy, its level can even reach almost 160 nmol/L.^88,110^

Other studies also used the LC–MS/MS method for the determination of steroids. Zhang et al. developed and validated a method based on UHPLC–MS/MS for the analysis of selected endogenous and synthetic estrogens and progestins in serum.^49^ Unlike the method described here, in this case, more than three times the volume of human serum is used. Even in other cases, the sample consumption is several times higher.^22,43,45,47^ When small sample volumes are used, the sample preparation for the analysis is usually more time-consuming and involves, for example, protein precipitation, LLE, SPE, and derivatization steps.^34^ Compared to other methods used for the steroid analysis, the described method works with a very small sample volume (150 μL) and does not require any specific purification techniques or chemical derivatization. Other published methods also use such small volumes of blood serum (100 μL) for the steroid analysis.^41^ Yesildal et al. developed a method based on isotope dilution UHPLC–MS/MS to profile a panel of steroids most commonly analyzed in clinical laboratories (aldosterone, corticosterone, cortisol, cortisone, 11-deoxycortisol, ANDRO, DHEA, dehydroepiandrosterone sulfate, DHT, estradiol, 17α-hydroxy progesterone, PROG, and T). Sample preparation also involves only a precipitation step by precipitant solution (ISs, zinc sulfate solution, and MeOH), but the sample injection for the analysis is 25 μL. In the case of our method, a small injection volume (only 2 μL) allows a reanalysis of the sample. Our method allows for simultaneous profiling of endogenous levels of progestin and androgen representatives in human blood serum, which was confirmed on a set of volunteer samples (different age and sex). Due to its reliability and simplicity, this method could be used in epidemiological studies. In addition, the discovery of reliable serum biomarkers is still a major challenge.^60^

## 3 Conclusions

Our research presents a novel complex method for the determination of selected NASs, including four progestins (PREG, PROG, ALLO, and DHP) and five androgens (DHEA, T, DHT, ANDRO, and EPIA) in human serum within one analytical run. Unlike the collection of the cerebrospinal fluid, obtaining blood serum is relatively easy and less invasive and stressful. Therefore, the discovery of new biomarkers of neurodegenerative diseases in this type of sample would bring about considerable advantages. Our developed and validated method using very small sample and injection volumes has many potential applications. To illustrate, it can serve as a tool for monitoring the differences between the levels of steroid hormones under different physiological or pathological conditions. The demonstrated method can be an ideal instrument for finding new biomarkers useful in the prevention, diagnosis, or monitoring of conditions associated with changes in NAS levels for a better understanding of the pathophysiology of certain diseases, as well as for discovering new drugs or developing new therapeutic approaches.

## 4 Materials and Methods

### 4.1 Human and Animal Rights Statement

The use of human serum samples was approved by the institutional ethics committee of the Faculty of Medicine and Dentistry, Palacky University in Olomouc and University Hospital Olomouc. Written informed consent was obtained from all participants.

### 4.2 Chemicals and Materials

Unlabeled standards PREG, ALLO, PROG, ANDRO, and DHP were purchased from Sigma-Aldrich (Germany). The T standard was obtained from Fluka (Netherlands), and DHEA and DHT were from the National Measurement Institute (Australia). EPIA was prepared by palladium-catalyzed hydrogenation of DHEA according to a published procedure.^115^ Internal standards (ISs) labeled with deuterium pregnenolone-*d*_4_ (*d*_4_-PREG), allopregnanolone-*d*_4_ (*d*_4_-ALLO), and *d*_9_-PROG were obtained from Cambridge Isotope Laboratories, Inc. (USA). Testosterone-*d*_3_ (*d*_3_-T) and dehydroepiandrosterone-*d*_6_ (*d*_6_-DHEA) were purchased from Sigma-Aldrich (USA). All stocks and working solutions of standards (ISs, unlabeled standards) were dissolved in 100% MeOH and stored in the dark at −80 °C until analysis.

The solvents MeOH gradient-grade for LC, MeOH hypergrade for LC–MS, and acetonitrile (ACN) hypergrade for LC–MS were purchased from Merck Millipore (Germany). Pure water was prepared using a Direct-Q 3 UV water purification system (Merck Millipore, Germany). Formic acid was obtained from Fluka (USA), and butylated hydroxytoluene and bovine serum albumin were obtained from Sigma-Aldrich (USA). All other chemicals used were purchased from Lachner (Czech Republic).

Method calibration was performed using a surrogate matrix prepared by dissolution of 4% bovine serum albumin in 10 mmol/L PBS, pH 7.4. The PBS buffer was composed of 136.9 mmol/L sodium chloride, 2.7 mmol/L potassium chloride, 10.1 mmol/L disodium phosphate dodecahydrate, and 1.8 mmol/L monopotassium phosphate. The surrogate matrix was aliquoted and stored in the dark at −80 °C. A new aliquot of the surrogate matrix was used for each experiment.

### 4.3 Sample Collection, Pretreatment, and Storage

Human serum samples required for the development and validation of the method were obtained from the Department of Neurology of the University Hospital Olomouc, Czech Republic. Ethics approval was granted according to the University Hospital Olomouc standard SM-L031 and ethics committee reference numbers 139/10 and 76/15. The collection of blood samples from participants and their pretreatment, transport, and storage were performed according to the established methodologies.

Peripheral blood was collected at 10:00 a.m. with a prior 18 h fasting period by venipuncture into sterile collection tubes (VACUETTE 9 mL Z serum separator clot activator) and centrifuged at 4000 rpm and 4 °C for 5 min (Universal 320R, Hettich, Germany). The obtained sera were transferred to dark amber glass vials and treated in an ultrasonic bath for 5 min (Elmasonic S 10 H, Elma, Germany). Serum samples in vials were then bubbled with a stream of argon for 2 min to create an inert atmosphere. Argon provides prevention against the unwanted oxidation of analytes. Finally, these samples were stored in the dark at −80 °C until analysis. Due to the distinct primary purpose of sample collection, we lack further details on treatment, women’s menstrual or reproductive status, etc.

### 4.4 Serum Sample Processing

**Figure 4:**
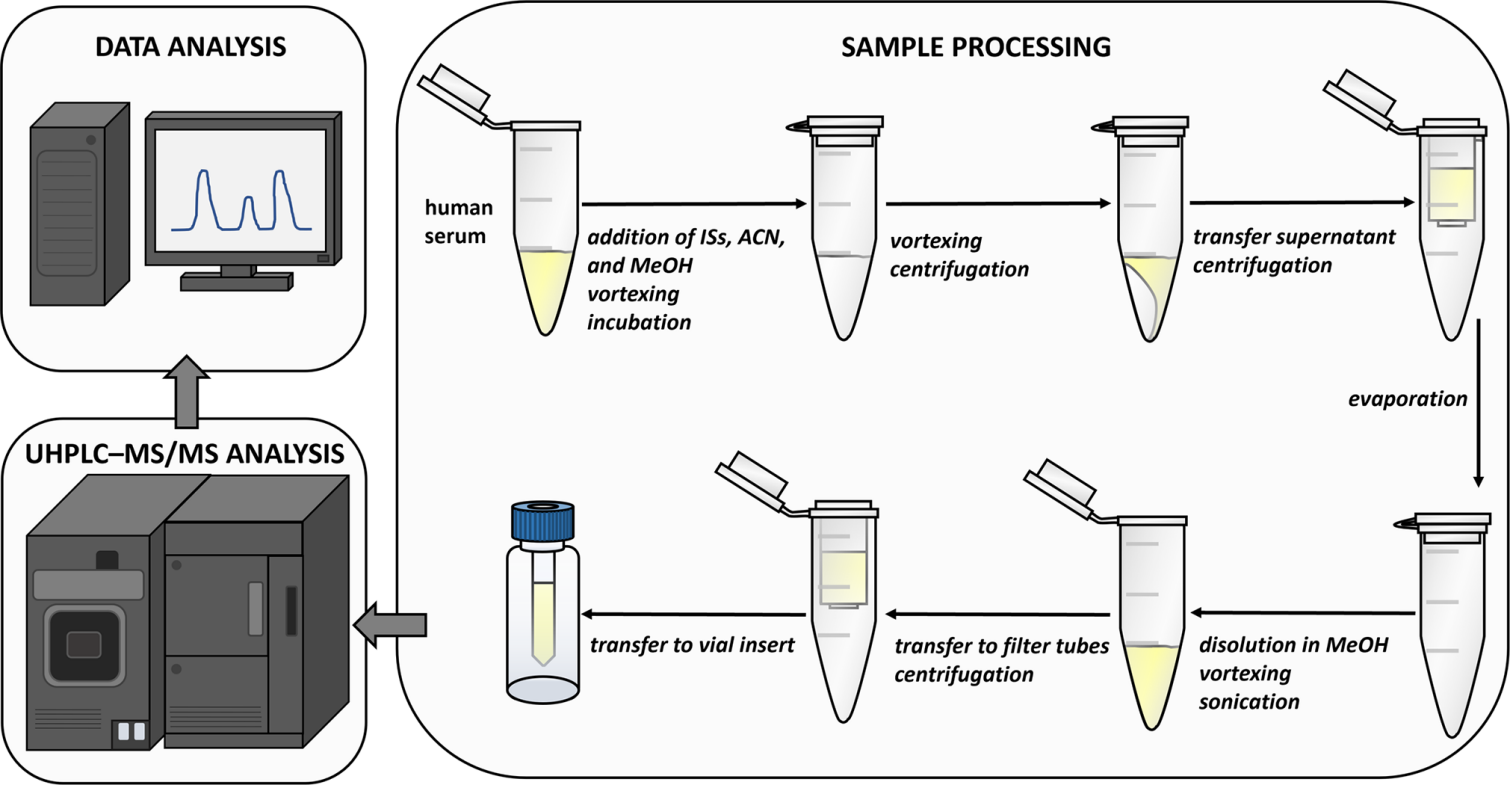
Main steps of the developed method for profiling selected neuroactive steroids in serum: sample processing, UHPLC–MS/MS analysis, and data analysis. UHPLC–MS/MS: ultrahigh-performance liquid chromatography–tandem mass spectrometry, ISs: internal standards, ACN: acetonitrile, and MeOH: methanol.

First, 5 μL of a stock solution containing a mixture of stable isotopically labeled ISs (addition of IS mixture A) was added to 150 μL of cooled human serum. A list of ISs and their additions is provided in Table 8. The modified sample preparation was based on a previously published protocol.^116^ Briefly, serum proteins were completely precipitated by adding 595 μL of ice-cold ACN (−20 °C) containing 0.05% (v/v) butylated hydroxytoluene (prevention of autoxidation).^117^ The addition of ACN ensures both the precipitation of serum proteins and the release of steroid substances from their carrier proteins. Finally, 45 μL of MeOH was also added, corresponding to the addition of steroid standards in the calibration curve samples. Serum samples were kept cold during all pipetting steps (CoolBox, Biocision, USA). The resulting precipitate was vortexed for 30 s (Wizard Advanced IR Vortex Mixer, VELP Scientifica, Italy). The samples were placed on a rotator (SB3, Stuart, UK) and incubated for 60 min at 19 rpm at −20 °C to ensure protein precipitation. After further vortexing (30 s), the samples were centrifuged at 10,000 rpm for 10 min at 4 °C (Heraeus Multifuge X1R, Thermo Scientific, USA). The supernatant obtained was transferred to a minispin centrifuge filter tube with a nylon membrane and a pore size of 0.20 μm (Mini Spin Columns +0.2 NY). The samples were filtered at 10,000 rpm for 5 min and 4 °C. The filtrate was then evaporated to dryness under a gentle stream of nitrogen at 37 °C for as long as necessary (TurboVap Classic LV, Biotage, Sweden). The dry residues were then dissolved in 50 μL of 100% ice-cold MeOH, vortexed to rinse the microtube walls (30 s), and placed in an ultrasonic bath (3 min) (Sonorex RK 510S, Bandelin, Germany). The dissolved samples were then transferred to a minispin centrifuge filter tube, centrifuged at 10,000 rpm for 3 min at 4 °C, and pipetted into vial inserts for LC–MS measurements. The processed serum samples in triplicates were then immediately placed in the cooled autosampler (4 °C) of the UHPLC–MS/MS instrument and analyzed. The dilution factor for all serum samples was 1/3 (150 μL of serum was precipitated, evaporated to dryness, and then dissolved in 50 μL of methanol prior to injection into the column). A schematic overview of the main steps in the analysis of NASs of interest is shown in Figure 4.

**Table 8:** Overview of Individual Analytes, the Corresponding Deuterated Internal Standards, and Their Additions^a^

		**calibration range** (nmol/L)	
		0.09–90.00	284.60–28,460.50
**analyte**	**IS**	**addition IS mix A** (nmol/L)	**addition IS mix B** (nmol/L)
DHEA	*d*_6_-DHEA	100	1000
T	*d*_3_-T	10	100
EPIA	*d*_3_-T	10	100
DHT	*d*_3_-T	10	100
ANDRO	*d*_3_-T	10	100
PREG	*d*_4_-PREG	500	5000
ALLO	*d*_4_-ALLO	1000	10,000
PROG	*d*_9_-PROG	10	100
DHP	*d*_9_-PROG	10	100

a ALLO: allopregnanolone, ANDRO: androstenedione, *d*_3_-T: testosterone-*d*_3_, *d*_4_-ALLO: allopregnanolone-*d*_4_, *d*_4_-PREG: pregnenolone-*d*_4_, *d*_6_-DHEA: dehydroepiandrosterone-*d*_6_, *d*_9_-PROG: progesterone-*d*_9_, DHEA: dehydroepiandrosterone, DHP: 5α-dihydroprogesterone, DHT: 5α-dihydrotestosterone, EPIA: epiandrosterone, PREG: pregnenolone, PROG: progesterone, IS: internal standard, and T: testosterone.

### 4.5 Chromatographic Separation and Mass Spectrometric Detection

The UHPLC–MS/MS analysis of targeted steroid compounds was performed using an ACQUITY UPLC H-Class PLUS system (Waters, USA) connected to a triple-quadrupole MS Xevo TQ-S micro (Waters, UK) with an ESI source. Data acquisition and processing were performed using MassLynx 4.1 (Waters) and Microsoft Office (Microsoft) software packages.

A Kinetex biphenyl column (100 × 2.1 mm, 1.7 μm, 100 Å; Phenomenex, USA) equipped with an ACQUITY column in-line filter kit (Waters) was used for the chromatographic separation of steroid compounds. The column was maintained at 40 °C with a flow rate of 0.5 mL/min of the mobile phase containing 100% MeOH (A) and 7.5 mmol/L aqueous solution of formic acid (B). The gradient was as follows: 0 min, 60:40 (A/B); 10 min, 75:25 (A/B); 12 min, 85:15 (A:B); 12.25–12.75 min, 99:1 (A:B); 13–15 min, 60:40 (A/B). Wash and equilibration steps were included at the end of the gradient used. The column was washed with 99% MeOH for 0.50 min. After the washing step, initial separation conditions were achieved using a 2 min equilibration. The course of the gradient is shown in Figure 2. The mobile phase was directed into the MS from 3.51 min of the gradient. Thus, only at the moment of the expected elution of the first analytes, this prevented unnecessary fouling of the MS instrument components. Similarly, the mobile phase flow was directed to the waste at the end of the gradient (11.49 min). During the UHPLC–MS/MS analysis, samples were placed in an autosampler maintained at 4 °C in the dark. Samples dissolved in 100% MeOH were injected (constant injection volume of 2 μL) into a reverse phase column. The representative chromatographic separation of the steroid standards in 100% MeOH and their RTs are shown in Figure 2. The chromatographic column was washed at the end of the analysis and stored for a long time in 65% ACN in water. The total run time was 15 min per sample.

A triple-quadrupole MS instrument was operated in the positive ESI mode by using MRM transitions. The optimized conditions for the MS analysis were the following: a source temperature of 150 °C, a desolvation temperature of 600 °C, a desolvation nitrogen gas flow rate of 1000 L/h, a capillary voltage of 2.5 kV, a cone voltage of 19–45 V, and a collision energy of 11–30 eV. A specific quantification and confirmation of MRM transitions were selected for each steroid analyte. An MRM transition was also selected for each IS. In addition, based on the RT knowledge of the individual analytes, the MS/MS measurement was divided into five separate MRM scan segments. In these short RT windows, only the mass transitions of the expected analytes were measured. The dwell times were determined based on the width of the chromatographic peak. The dwell time value was set on the MS so that the obtained chromatographic peaks were covered by at least 15 scan points. The MRM transitions and other selected MS parameters for individual analytes and the corresponding ISs are listed in Table 2.

### 4.6 Bioanalytical Method Validation

Validation is a tool that can be used to assess whether a bioanalytical method is suitable for its intended purpose. The parameters that should be verified during validation and the criteria that the method should meet are described in detail in the EMA^86^ and Food and Drug Administration (FDA)^118^ guidelines. Four QC levels of the analyte concentration were used for the following series of validation experiments, namely, the LQ, MQ, HQ, and UHQ levels. The QC levels were chosen to cover the linear calibration range of each analyte with respect to endogenous serum steroid levels. The LQ, MQ, HQ, and UHQ levels correspond to 28.46, 90, 900, and 2846.05 nmol/L, respectively.

### 4.7 Method Calibration

When analyzing metabolites in biological matrices, it is recommended to prepare calibration points in the same, usually artificial matrix.^86^ Matrix surrogate calibration curves prepared from 4% bovine serum albumin solution in a 10 mmol/L PBS buffer were used the for quantification of steroid analytes.^116^ This surrogate matrix replaces real human serum. Each calibration point (prepared in triplicate) contained a surrogate matrix (150 μL), a mixture of unlabeled standards (45 μL), a defined addition of stable isotopically labeled ISs (5 μL, addition of IS mixture A or B) (Table 8), and 100% ice-cold ACN (595 μL, −20 °C). The calibration range was divided into two parts to obtain the optimal matrix surrogate calibration curves. Each part of the calibration points contained a mixture of ISs with different concentrations (addition of IS mixture A or B). Table 8 shows both parts of the calibration range and the optimized IS additions. The same extraction protocol was applied to the calibration samples in a surrogate matrix as well as to the real blood serum samples (based on the procedure described in the “Serum Sample Processing” section). No weighting factor was used for calibration.

Finally, the defined addition of ISs allows the quantification of endogenous analytes in unknown samples using the isotope dilution method.^80^ This method is based on knowing the ratio between the area of the analyte and the labeled standard in the sample (the so-called response), which is then plotted on a calibration curve. The result of this interpolation is absolute quantification of the analyte in the sample.

### 4.8 Method Precision and Accuracy

Within-run precision and accuracy were determined using four sets of neat solution samples (100% MeOH) spiked with a constant amount of labeled ISs (addition of IS mixture A or B) and unlabeled standards at the LQ, MQ, HQ, or UHQ levels. The samples were analyzed in five replicates for each QC level. The UHPLC–MS/MS analysis of the prepared samples was performed within one run of the instrument. The same sample sets of neat solutions were used to determine the between-run precision and accuracy of the method. These were analyzed for each concentration level (LQ, MQ, HQ, and UHQ) in five replicates on three different runs on two different days.

Within-run precision and accuracy were also determined using the donor pool serum. The serum was divided into four sets based on the addition of unlabeled standards. All sets were spiked with a constant addition of ISs (addition of IS mixture A or B) and unlabeled analytes at the LQ, MQ, HQ, or UHQ level. The samples were analyzed within one run of the instrument.

### 4.9 Method Recovery and Matrix Effects

Four individual human serum donors were selected to determine the analytical RE and ME. These validation parameters were determined for each analyte at four concentration levels. The LQ, MQ, HQ, and UHQ levels correspond to 28.46, 90, 900, and 2846.05 nmol/L, respectively. To establish appropriate QC concentration levels, the endogenous levels of the analytes of interest in the human serum were preliminarily measured. The first set of serum samples was spiked with a mixture of unlabeled standards to four concentration levels and a constant amount of ISs (addition of IS mixture A or B) at the beginning of the extraction protocol. Individual QC levels were represented by samples in triplicate. The same extraction protocol described in the “Serum Sample Processing” section was applied to the prepared samples. At the same time, the other corresponding set of samples was prepared and spiked with standards after extraction prior to the UHPLC–MS/MS analysis. Adequate blanks (individual donor serum samples that were spiked with a mixture of ISs before or after extraction) were also prepared for both sets of samples to subtract endogenous analyte levels.

The RE (eq 1) of the analytical method was calculated from the mean peak area of the analyte in the matrix spiked with standards before extraction with subtraction of the area of endogenous analyte levels (member C) to the mean peak area of the analyte in the matrix spiked after extraction with subtraction of the area of endogenous analyte levels (member B). The RE values were calculated based on the following previously published equations.^97^
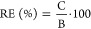
1

The ME (eq 2) was calculated by knowing the ratio of the mean peak area of the analyte in the matrix spiked with standards after extraction with subtraction of the area of endogenous analyte levels (member B) to the mean peak area in the neat solution of the analyte without the presence of a matrix (member A).^97,119^ The resulting ME is reported as a percentage. An ME value greater than 100% reflects an enhancement of ionization, and a value less than 100% indicates a suppression of ionization. It was determined based on the following calculation:
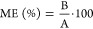
2

Furthermore, the so-called IS-normalized ME (eq 3) was also determined.^100^ As in the previous case, this is also a postextraction addition technique for ME evaluation. Its calculation is based on the ratio of the response in the matrix with subtraction of the response of endogenous analyte levels (member D; spiked after extraction) to the response in the neat solution (member E). The response is determined as the ratio between the peak area of the analyte and the IS. The IS-normalized ME was expressed by the equation
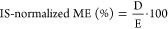
3

### 4.10 Stability of the Analytes

The stability of the steroid analytes was assessed under autosampler storage conditions at 4 °C for 7 days. Neat solutions, spiked pooled serum, and surrogate matrix at three concentration levels (LQ, MQ, and UHQ levels correspond to 28.46, 90, and 2846.05 nmol/L) were used for this evaluation. All samples were prepared for each concentration level in triplicate and then stored in an autosampler at 4 °C prior to injection. The specific time points evaluated were days 0, 1, 3, and 7. Analyte stability determination was based on monitoring changes in the analyte to IS area ratio at each time point compared with the ratio measured at the initial time point (day 0).

## Data Availability

The data sets generated during and/or analyzed during the current study are available from the corresponding author on reasonable request.

## Supporting Information Available

Table S1 showing physicochemical properties of the analytes; Tables S2, S3, and S4 listing the stability results of steroid analytes (PDF)

## Notes

Ethics approval for this study was granted according to University Hospital Olomouc standard SM-L031 and ethics committee reference numbers 139/10 and 76/15.

## Abbreviations

DHP: 5α-dihydroprogesterone
DHT: 5α-dihydrotestosterone
ACN: acetonitrile
ALLO: allopregnanolone
ANDRO: androstenedione
APCI: atmospheric-pressure chemical ionization
APPI: atmospheric-pressure photoionization
CV: coefficient of variation
*d*_3_-T: testosterone-*d*_3_
*d*_4_-ALLO: allopregnanolone-*d*_4_
*d*_4_-PREG: pregnenolone-*d*_4_
*d*_6_-DHEA: dehydroepiandrosterone-*d*_6_
*d*_9_-PROG: progesterone-*d*_9_
DHEA: dehydroepiandrosterone
EMA: European Medicine Agency
EPIA: epiandrosterone
ESI: electrospray ionization
FDA: Food and Drug Administration
F: female
GC: gas chromatography
HQ: high-quality control
IS: internal standard
LC: liquid chromatography
LLE: liquid–liquid extraction
LLOQ: lower limit of quantification
LOD: limit of detection
LQ: low-quality control
M: male
ME: matrix effect
MeOH: methanol
MQ: medium-quality control
MRM: multiple-reaction monitoring
MS: mass spectrometry
MS/MS: tandem mass spectrometry
MW: molecular weight
NAS: neuroactive steroid
PBS: phosphate-buffered saline
PREG: pregnenolone
PROG: progesterone
QC: quality control
RE: recovery
RIA: radioimmunoassay
RT: retention time
SPE: solid-phase extraction
SD: standard deviation
T: testosterone
UHPLC–ESI–MS/MS: ultrahigh-performance liquid chromatography with electrospray tandem mass spectrometry
UHQ: ultrahigh-quality control
ULOQ: upper limit of quantification
