# Supplementary material for: Simultaneous Determination of Selected Steroids with Neuroactive Effects in Human Serum by Ultrahigh-Performance Liquid Chromatography–Tandem Mass Spectrometry
Source: ACS Chem Neurosci. 2024 Apr 24;15(10):1990–2005. doi: 10.1021/acschemneuro.3c00824 (PMC11099924; doi:10.1021/acschemneuro.3c00824)
Supplement: Supplementary file 1 — cn3c00824_si_001.pdf [file cn3c00824_si_001.pdf]

ACS Chemical Neuroscience

Supporting Information

Simultaneous Determination of Selected Steroids with Neuroactive Effects in Human Serum by Ultra-High Performance Liquid Chromatography–Tandem Mass Spectrometry

Michal Kaleta[a,b*], Jana Oklestkova[a], Kateřina Klíčová[b,c], Miroslav Kvasnica[a], Dorota Koníčková[b,c], Kateřina Menšíková[b,c], Miroslav Strnad[a], Ondřej Novák[a*]

[a]Laboratory of Growth Regulators, Faculty of Science, Palacký University & Institute of Experimental Botany of the Czech Academy of Sciences, Šlechtitelů 27, 783 71 Olomouc, Czech Republic

[b]Department of Neurology, Faculty of Medicine and Dentistry, Palacký University, 779 00 Olomouc, Czech Republic

[c]Department of Neurology, University Hospital Olomouc, 779 00 Olomouc, Czech Republic

Correspondence*

Ondřej Novák
E-mail: novako@ueb.cas.cz

Michal Kaleta
E-mail: michal.kaleta@upol.cz

Table S1. Selected physico-chemical properties of the analytes (data source used HMBD: Human Metabolome Database [1]).

Analyte	Log P	pKa_1 (strongest acidic)	pKa_2 (strongest basic)	Hydrogen acceptor count	Hydrogen donor count
DHEA	3.36	18.20	-1.40	2	1
T	3.37	19.09	-0.88	2	1
EPIA	3.77	18.30	-1.40	2	1
DHT	3.41	19.38	-0.88	2	1
ANDRO	3.93	19.03	-4.80	2	0
PREG	3.58	18.20	-1.40	2	1
ALLO	3.99	18.30	-1.40	2	1
PROG	4.15	18.92	-4.80	2	0
DHP	4.19	19.34	-7.10	2	0

ALLO: Allopregnanolone, ANDRO: Androstenedione, DHEA: Dehydroepiandrosterone, DHP: α-Dihydroprogesterone, DHT: α-Dihydrotestosterone, EPIA: Epiandrosterone, PREG: Pregnenolone, PROG: Progesterone, T: Testosterone.

References

(1) Wishart, D. S.; Guo, A.; Oler, E.; Wang, F.; Anjum, A.; Peters, H.; Dizon, R.; Sayeeda, Z.; Tian, S.; Lee, B. L.; Berjanskii, M.; Mah, R.; Yamamoto, M.; Jovel, J.; Torres-Calzada, C.; Hiebert-Giesbrecht, M.; Lui, V. W.; Varshavi, D.; Varshavi, D.; Allen, D.; Arndt, D.; Khetarpal, N.; Sivakumaran, A.; Harford, K.; Sanford, S.; Yee, K.; Cao, X.; Budinski, Z.; Liigand, J.; Zhang, L.; Zheng, J.; Mandal, R.; Karu, N.; Dambrova, M.; Schiöth, H. B.; Greiner, R.; Gautam, V. HMDB 5.0: The Human Metabolome Database for 2022. *Nucleic Acids Res* **2022**, *50*, 622–631.

Table S2. Stability of steroid analytes in neat solutions at low (LQ), medium (MQ), and ultra-high (UHQ) levels during storage in an autosampler at 4 °C (*n* = 3).

	Stability of analytes in neat solutions mean (%) (SD)								
**QC levels**[a]	**LQ**			**MQ**			**UHQ**		
	**day 1**	**day 3**	**day 7**	**day 1**	**day 3**	**day 7**	**day 1**	**day 3**	**day 7**
DHEA	103 (3.9)	101 (2.9)	104 (5.5)	101 (0.7)	100 (3.0)	102 (1.8)	101 (0.4)	100 (1.4)	101 (0.9)
T	100 (2.8)	105 (1.9)	101 (1.9)	98 (0.3)	103 (2.3)	99 (1.6)	97 (2.0)	103 (1.9)	98 (1.3)
EPIA	103 (2.4)	102 (1.1)	99 (0.9)	98 (2.3)	101 (0.3)	97 (1.7)	98 (1.5)	104 (1.4)	102 (0.7)
DHT	103 (3.7)	109 (1.0)	101 (2.2)	100 (1.4)	108 (3.3)	99 (2.4)	98 (0.8)	109 (1.4)	102 (0.2)
ANDRO	102 (2.7)	108 (1.5)	102 (0.7)	100 (1.2)	107 (3.5)	100 (1.3)	97 (1.4)	107 (1.8)	101 (0.4)
PREG	102 (2.6)	96 (4.2)	102 (9)	98 (3.8)	93 (3.1)	100 (3.2)	101 (2.2)	94 (0.3)	101 (2.5)
ALLO	101 (2.8)	99 (3.7)	99 (3.7)	101 (3.5)	101 (2.7)	103 (2.7)	101 (2.3)	101 (2.1)	102 (0.6)
PROG	100 (1.6)	101 (0.6)	102 (1.3)	100 (1.4)	101 (1.5)	102 (1.7)	100 (0.7)	100 (1.5)	97 (0.1)
DHP	97 (0.9)	104 (1.7)	98 (2.9)	98 (2.1)	101 (1.2)	96 (2.3)	99 (0.4)	101 (1.0)	95 (0.9)

ALLO: Allopregnanolone, ANDRO: Androstenedione, DHEA: Dehydroepiandrosterone, DHP: α-Dihydroprogesterone, DHT: α-Dihydrotestosterone, EPIA: Epiandrosterone, PREG: Pregnenolone, PROG: Progesterone, T: Testosterone.
[a]The low (LQ), medium (MQ), and ultra-high (UHQ) levels correspond to 28.46, 90, and 2846.05 nmol/L, respectively.

Table S3. Stability of steroid analytes in pooled serum at low (LQ), medium (MQ), and ultra-high (UHQ) levels during storage in an autosampler at 4 °C (*n* = 3).

	Stability of analytes in pooled serum mean (%) (SD)								
**QC levels**[a]	**LQ**			**MQ**			**UHQ**		
	**day 1**	**day 3**	**day 7**	**day 1**	**day 3**	**day 7**	**day 1**	**day 3**	**day 7**
DHEA	100 (3.1)	103 (3.3)	108 (5.6)	104 (2.1)	102 (4.0)	104 (3.2)	100 (0.9)	101 (2.1)	99 (1.3)
T	98 (1.3)	101 (2.7)	96 (0.8)	96 (1.3)	100 (3.2)	96 (1.9)	96 (1.6)	98 (1.5)	94 (1.8)
EPIA	96 (3.6)	103 (2.3)	104 (0.6)	100 (1.6)	105 (1.1)	107 (1.4)	100 (0.7)	105 (0.1)	108 (0.3)
DHT	97 (5.0)	114 (2.1)	112 (0.6)	100 (2.1)	118 (4.1)	118 (2.5)	101 (2.6)	118 (1.4)	121 (2.6)
ANDRO	96 (1.8)	100 (1.1)	97 (3.6)	96 (3.0)	102 (0.6)	98 (2.1)	97 (2.3)	100 (1.2)	99 (1.1)
PREG	102 (2.0)	96 (3.9)	105 (5.3)	102 (1.8)	98 (2.3)	100 (1.4)	104 (1.6)	97 (1.4)	107 (2.0)
ALLO	95 (7.0)	107 (14.1)	114 (11.7)	101 (1.8)	108 (2.6)	111 (1.8)	100 (0.6)	110 (2.3)	115 (0.7)
PROG	104 (1.6)	98 (1.5)	89 (3.5)	105 (1.5)	100 (1.7)	90 (3.7)	105 (1.5)	100 (3.3)	88 (5.8)
DHP	115 (3.9)	137 (4.6)	160 (2.7)	108 (0.6)	125 (1.6)	130 (1.6)	106 (1.1)	119 (0.5)	123 (0.6)

ALLO: Allopregnanolone, ANDRO: Androstenedione, DHEA: Dehydroepiandrosterone, DHP: α-Dihydroprogesterone, DHT: α-Dihydrotestosterone, EPIA: Epiandrosterone, PREG: Pregnenolone, PROG: Progesterone, T: Testosterone.
[a]The low (LQ), medium (MQ), and ultra-high (UHQ) levels correspond to 28.46, 90, and 2846.05 nmol/L, respectively.

Table S4. Stability of steroid analytes in surrogate matrix at low (LQ), medium (MQ), and ultra-high (UHQ) levels during storage in an autosampler at 4 °C (n = 3).

	Stability of analytes in surrogate matrix mean (%) (SD)								
**QC levels**[a]	**LQ**			**MQ**			**UHQ**		
	**day 1**	**day 3**	**day 7**	**day 1**	**day 3**	**day 7**	**day 1**	**day 3**	**day 7**
DHEA	104 (2.5)	106 (0.5)	105 (5.1)	99 (3.2)	101 (4.1)	101 (3.2)	99 (1.1)	100 (0.6)	98 (0.4)
T	97 (1.0)	101 (0.9)	97 (0.6)	99 (2.8)	100 (0.7)	98 (1.0)	98 (2.1)	101 (2.4)	98 (0.6)
EPIA	99 (1.7)	102 (0.8)	98 (2.9)	100 (2.5)	101 (0.4)	99 (1.3)	99 (2.4)	103 (1.7)	107 (0.9)
DHT	99 (0.5)	105 (3.0)	96 (1.7)	100 (3.0)	104 (1.6)	100 (0.5)	99 (2.2)	105 (2.1)	106 (0.5)
ANDRO	98 (0.9)	105 (1.2)	102 (2.9)	100 (2.8)	104 (1.8)	103 (0.6)	98 (1.6)	105 (1.4)	106 (0.9)
PREG	104 (0.1)	96 (0.1)	106 (2.3)	101 (1.3)	95 (3.5)	100 (4.5)	101 (2.6)	95 (1.3)	102 (0.7)
ALLO	104 (7.9)	103 (5.2)	103 (5.1)	100 (1.5)	101 (1.5)	101 (1.5)	101 (0.7)	101 (1.1)	103 (1.9)
PROG	100 (1.0)	101 (1.5)	103 (1.0)	101 (0.1)	102 (2.1)	102 (0.5)	100 (0.1)	98 (1.0)	95 (1.2)
DHP	98 (2.6)	92 (2.4)	91 (2.4)	99 (1.5)	96 (1.7)	90 (2.0)	98 (0.6)	96 (1.6)	90 (1.5)

ALLO: Allopregnanolone, ANDRO: Androstenedione, DHEA: Dehydroepiandrosterone, DHP: α-Dihydroprogesterone, DHT: α-Dihydrotestosterone, EPIA: Epiandrosterone, PREG: Pregnenolone, PROG: Progesterone, T: Testosterone.

[a]The low (LQ), medium (MQ), and ultra-high (UHQ) levels correspond to 28.46, 90, and 2846.05 nmol/L, respectively.
